# Simple Surface Modification of Poly(dimethylsiloxane) via Surface Segregating Smart Polymers for Biomicrofluidics

**DOI:** 10.1038/s41598-019-43625-5

**Published:** 2019-05-14

**Authors:** Aslıhan Gökaltun, Young Bok (Abraham) Kang, Martin L. Yarmush, O. Berk Usta, Ayse Asatekin

**Affiliations:** 1 Center for Engineering in Medicine at Massachusetts General Hospital, Harvard Medical School, and Shriners Hospital for Children, 51 Blossom St., Boston, MA 02114 USA; 2 Department of Chemical and Biological Engineering, Tufts University, 4 Colby Street, Medford, MA 02474 USA; 3 Department of Chemical Engineering, Hacettepe University, 06532 Beytepe, Ankara Turkey; 4 Department of Biomedical Engineering, Rutgers University, 599 Taylor Rd., Piscataway, NJ 08854 USA

**Keywords:** Biosurfaces, Biomedical materials, Biomedical engineering, Biomedical materials, Assay systems

## Abstract

Poly(dimethylsiloxane) (PDMS) is likely the most popular material for microfluidic devices in lab-on-a-chip and other biomedical applications. However, the hydrophobicity of PDMS leads to non-specific adsorption of proteins and other molecules such as therapeutic drugs, limiting its broader use. Here, we introduce a simple method for preparing PDMS materials to improve hydrophilicity and decrease non-specific protein adsorption while retaining cellular biocompatibility, transparency, and good mechanical properties without the need for any post-cure surface treatment. This approach utilizes smart copolymers comprised of poly(ethylene glycol) (PEG) and PDMS segments (PDMS-PEG) that, when blended with PDMS during device manufacture, spontaneously segregate to surfaces in contact with aqueous solutions and reduce the hydrophobicity without any added manufacturing steps. PDMS-PEG-modified PDMS samples showed contact angles as low as 23.6° ± 1° and retained this hydrophilicity for at least twenty months. Their improved wettability was confirmed using capillary flow experiments. Modified devices exhibited considerably reduced non-specific adsorption of albumin, lysozyme, and immunoglobulin G. The modified PDMS was biocompatible, displaying no adverse effects when used in a simple liver-on-a-chip model using primary rat hepatocytes. This PDMS modification method can be further applied in analytical separations, biosensing, cell studies, and drug-related studies.

## Introduction

The microfluidics industry encompasses a $2–4 billion market^1,2^, expected to grow by ~18%/year to $10–20 billion by the 2020s^1^. Academic interest in this field is growing at a similarly fast pace, with the number of publications on microfluidics doubling every 15 months^2^. This growth is driven mainly by biomicrofluidics such as point of care devices, drug manufacturing micro-reactors, toxicity screening with organs-on-chips, and microneedles/pumps for drug delivery^3^. However, choosing the right materials is critical for avoiding artefacts and reduced sensitivity in biomedical and diagnostic applications, including those that can arise from the adsorption of compounds of interest onto surfaces.

Poly(dimethylsiloxane) (PDMS) and other silicone elastomers offer a range of favorable properties for biomicrofluidics applications, including: (1) simple fabrication by replica molding, (2) good mechanical properties, (3) excellent optical transparency from 240 to 1100 nm, (4) biocompatibility and non-toxicity, and (5) high gas permeability^4^. Despite these merits, the hydrophobicity of PDMS (water contact angle ~108° ± 7°)^5^ often limits its applications where solutions comprising of biological samples are concerned. The hydrophobicity of the PDMS surface results in undesired non-specific adsorption of proteins, which in turn affects analyte transport and reduces separation performance and detection sensitivity^6^. The hydrophobicity of PDMS microchannels also makes it difficult to introduce aqueous solutions or mixtures of aqueous and organic solutions^7^. Since most of the work in microfluidics relies on using polar liquids, this causes a significant obstacle in many applications. This has led many groups to develop approaches to render the PDMS surface hydrophilic and resistant to protein adsorption^8^. These strategies include the use of high-energy treatments in the form of O_2_ plasma, UV/ozone treatments, and corona discharges to oxidize PDMS surfaces and to introduce alkoxy- or chloro-silanes for surface functionalization later on, coating of PDMS surfaces with polar functionalities using charged surfactants, polyelectrolyte multilayers (PEMs), chemical vapor deposition, silanization, phospholipid bilayers, and more recently, by attaching hydrophilic polymer brushes to the surface of PDMS via grafting-from and grafting-to approaches, hydrosilylation and click chemistry^8^.

While these interventions have proved successful in improving surface hydrophilicity, their broader use was often limited by chemical stability, the need for special equipment and/or hazardous routes^9^, and/or the length and complexity of their process for fabrication that is restrictive for large-scale implementation. In addition, many of these methods lead to loss of transparency, change in mechanical properties, surface cracking and increased roughness^10,11^. Finally, most of these methods do not provide a hydrophilic surface long term. Due to the mobility of PDMS chains, the surface becomes hydrophobic again over time, negating the initial benefits of treatment^12,13^ (Table 1). These issues curtail the benefits of these PDMS surface modification methods and emphasize the need for a new approach.

**Table 1:** Comparison of PDMS modification strategies with the approach described in this manuscript, the addition of a PDMS-PEG BCP with optimized processing.

Approach	WCA (°)	Throughput/Scalability	Shelf Life (Longevity)	Biocompatibility	Optical/mechanical effects
Plasma treatment^10,11^	50–60	Current process	Low (<3 days)	High	Cracking possible
Grafting-to (e.g. plasma, silanization)^28,58,59^	<10–100	Low-medium (2–7 added steps)	Medium (14–47 days)	Not reported	Likely not affected
Grafting-from (e.g. SI-ATRP)^60,61^	10–80	Very low (many added steps)	Medium-high (up to 3 months)	Not reported	Likely not affected
Physisorption^62–65^	15–90	Low (one-many added steps)	Typically low	Not reported	Likely not affected
Past studies with block copolymer (BCP) addition or other prepolymer additives^20–22^	63–104	Very high; no added steps	Not reported; BCP or other additives dependent	Not reported	Reduced optical clarity for low WCA in studies reported to date
A past study using PDMS-*b*-PEO addition^23^	21.5–80.9	Very high; no added steps	2 months	Not reported	Compromised mechanical properties at higher concentrations that yield hydrophilic surfaces
Current study -Addition of a PDMS-PEG BCP with optimized processing	<10–20	Very high; no added steps	Very high (up to 20 months)	High	None when well-designed

An alternative approach for creating more hydrophilic and fouling-resistant surfaces involves the use of surface-segregating smart copolymers. In this approach, an amphiphilic copolymer additive is blended with the base polymer before the manufacture of the final component. The hydrophilic sections of the copolymer drive it to the polymer/water interface, leading to surface segregation. When successful, this results in increased surface hydrophilicity, but only minor changes in bulk properties. This approach has been previously used in other fields and base materials. For instance, it enabled the preparation of filtration membranes with excellent, complete fouling resistance made of polyacrylonitrile (PAN)^14–16^ and poly(vinylidene fluoride) (PVDF)^17^. It was also used to prevent non-specific adsorption and cell adhesion on poly(methyl methacrylate) (PMMA) surfaces^18,19^.

Similarly, the use of amphiphilic or hydrophilic additives to PDMS during the manufacture of devices can lead to improved hydrophilicity. This approach is simple, often requiring no additional steps. If designed well, it can potentially lead to mechanical and optical properties similar to unmodified PDMS. Yet, to our knowledge, there are only a few studies^20–25^ that have focused on functionalizing the PDMS surface through a pre-mixing method, where functional additives are added to the liquid PDMS pre-polymer before curing. In some cases, the objective of such studies was not to improve hydrophilicity but to introduce specific functional groups on the surface. For example, Zare *et al*.^24^ added a biotinylated phospholipid to PDMS prepolymer to enable protein immobilization. Another study introduced charged groups to PDMS microfluidic channels by adding undecylenic acid to the pre-polymer prior to curing^25^. This led to increased electroosmotic flow (EOF) in PDMS microchannels, improving the separation efficiency and reducing the peak broadening in PDMS microfluidic devices. In both studies, the use of the additive did not lead to any changes in surface hydrophobicity. Other researchers have tested additives to improve surface hydrophilicity (Table 1). For instance, Zhou *et al*. added vinyl-terminated polyethylene glycol (PEG) chains to PDMS before curing^22^, showing a slight decrease in the water contact angle (WCA) from 112° to about 78°, accompanied by improved resistance to non-specific adsorption of a protein. In another study, PDMS microchips were prepared via adding a poly(lactic acid)–poly(ethylene glycol) (PLA–PEG) amphiphilic diblock copolymer before curing^20^. These microchips exhibited reduced myoglobin adsorption, and slightly lower WCAs of 84° and 73° for 1.5 and 2% mass ratio of PLA–PEG to PDMS, respectively. The amphiphilic triblock copolymer Pluronic (PEG-b-poly(propylene oxide)-b-PEG) was also used as a similar additive^21^. Upon filling the PDMS microfluidic channel with water, Pluronic embedded in PDMS segregated towards the water/PDMS interface. The static contact angle of modified PDMS surface changed from 98.6° to 63° after soaking the sample in water for 24 hours, whereas that of the additive-free PDMS remained around 103°. Furthermore, thanks to the improved hydrophilicity, the modified surface exhibited lower non-specific adsorption of Immunoglobulin G (IgG) compared to native PDMS. However, the limited compatibility of the hydrophobic poly(propylene oxide) segments with PDMS can limit the success of this approach. Indeed, the researchers observed samples became cloudy with as little as 0.16% Pluronic. Furthermore, the Pluronic surfactant is water soluble, which led to some leaching during use. This may lead to the degradation of surface hydrophilicity in time, and affect cell viability.

An alternative copolymer additive that has better compatibility with PDMS and can be integrated into the PDMS network during the preparation of the microchip would be beneficial. There is only one preliminary study that utilizes a PDMS-based additive, a PDMS-PEG block copolymer. This study shows that the addition of this PDMS-PEG block copolymer improved surface hydrophilicity of PDMS when used at concentrations between 1–1.9% (w/w), reducing the contact angle to 21.5–80.9°^23^. These results indicate that PDMS-PEG copolymers may be a promising initial direction for improving the surface properties of PDMS, motivating the work described here. However, this study also leaves a lot of questions open regarding the effectiveness of PDMS-PEG additives in biomicrofluidic applications, and their true performance gains. Importantly, both this study and most others in the literature study surfaces prepared upon blending PDMS with the additive but do not account for the effect of common processes used in the manufacture of actual microfluidic devices such as plasma treatment on the eventual surface chemistry. This is a crucial short-coming for understanding the applicability of this work to practical systems. Furthermore, the work mentions compromised mechanical properties at high concentrations of this additive but does not quantify it. Importantly, no studies that utilize PDMS-based additives characterize non-specific protein adsorption, or how the surface hydrophilicity changes with time or upon exposure to common processes used in manufacturing microfluidic devices. Finally, none of the studies that focus on surface modification using additives, PDMS-based or not, tested their materials for biocompatibility. It should be noted that these additives are typically surfactants that are water-soluble and can cause cell rupture. Therefore, it is crucial to test any such new approaches for biocompatibility to ensure its usability in realistic systems in contact with cells, such as organs-on-chips.

In this study, we focus on a practical and simple approach to improve the hydrophilicity of PDMS surfaces by adding a PDMS-PEG block copolymer (BCP) to the PDMS prepolymer before curing at concentrations between 0.25–2%, with the rest of the device manufacture process being conducted with no further changes, enabling this surface modification approach to be directly plugged into existing protocols. While a similar copolymer was previously used to improve the hydrophilicity of PDMS surfaces^23^, the experiments reported did not accurately address several key questions relevant to optimizing the overall biomicrofluidic device manufacturing process or the use of these devices in realistic applications. These results also implied compromised optical and mechanical properties at additive concentrations needed to improve hydrophilicity. Here, we seek to holistically study the use of a similar PDMS-PEG copolymer as an additive, with a focus on building a comprehensive understanding of how the overall device manufacture process, including alcohol soak and plasma treatment steps and exposure to cells, affects the surface chemistry and performance of devices prepared by this approach. We also seek to understand the capabilities of this approach in biomicrofluidic device applications, including the creation of devices with high optical clarity and mechanical properties. Importantly, we show that by tuning these manufacturing parameters and leveraging manufacturing steps already used for biomicrofluidic devices, we can achieve significantly enhanced hydrophilicity that is stable over at least 20 months, longer than all past reports, including those using more complex methods (Table 1). Compared to other additives that have been explored to date^20–25^, the utilization of this PDMS-PEG block copolymer provides better compatibility between the additive and PDMS, keeping the device optically clear at concentrations up to 0.25%. Through dynamic water contact angle (WCA) measurements, we show the PDMS-PEG copolymer segregates to the surface when exposed to water/aqueous solutions, which renders the surface more hydrophilic than all past studies using additives (Table 1). This approach also reduces non-specific adsorption of proteins (albumin, lysozyme and immunoglobulin G), as indicated by both fluorescent protein adsorption experiments on slabs and by quantitative experiments on fabricated microfluidic devices. The PDMS monolith and PDMS segments in block copolymers interact through van der Waals and hydrophobic interactions that improve the stability of the PEG layer on the PDMS surface^26^. Furthermore, the PDMS chains in the BCP can potentially be cross-linked with the chains of the monolith during the plasma treatment stage, further improving the stability of the hydrophilic surface. Indeed, we show that the hydrophilicity of PDMS modified with this copolymer is retained for at least twenty months, longer than all past reports, even after exposure to isopropanol (IPA) soaking and plasma treatment, crucial manufacturing steps that were not considered in previous studies. Mechanical properties are preserved at PDMS-PEG concentrations up to 1.0%, whereas optical clarity is retained at concentrations up to 0.25%. Unlike previous publications, this is the first report where the biocompatibility of PDMS modified with PDMS-PEG BCP was tested by culturing primary rat hepatocytes in glass-(PDMS-PEG BCP modified) PDMS microfluidic tissue culture devices. The PDMS-PEG modified devices performed just as well as unmodified PDMS devices and presented no adverse effects. These results demonstrate that the addition of this PDMS-PEG BCP to PDMS before the manufacture and curing of biomicrofluidic devices results in a durable increase in hydrophilicity and resistance to non-specific adsorption without sacrificing mechanical properties, optical clarity, or biocompatibility. Therefore, this method promises to be a very simple, rapid, and cost-effective approach to generate hydrophilic and protein repellent PDMS elastomer for microfluidic devices as well as other uses such as tubing and sealants.

## Results and Discussion

### Surface modification of PDMS with PDMS-PEG BCP additives

**Figure 1:**
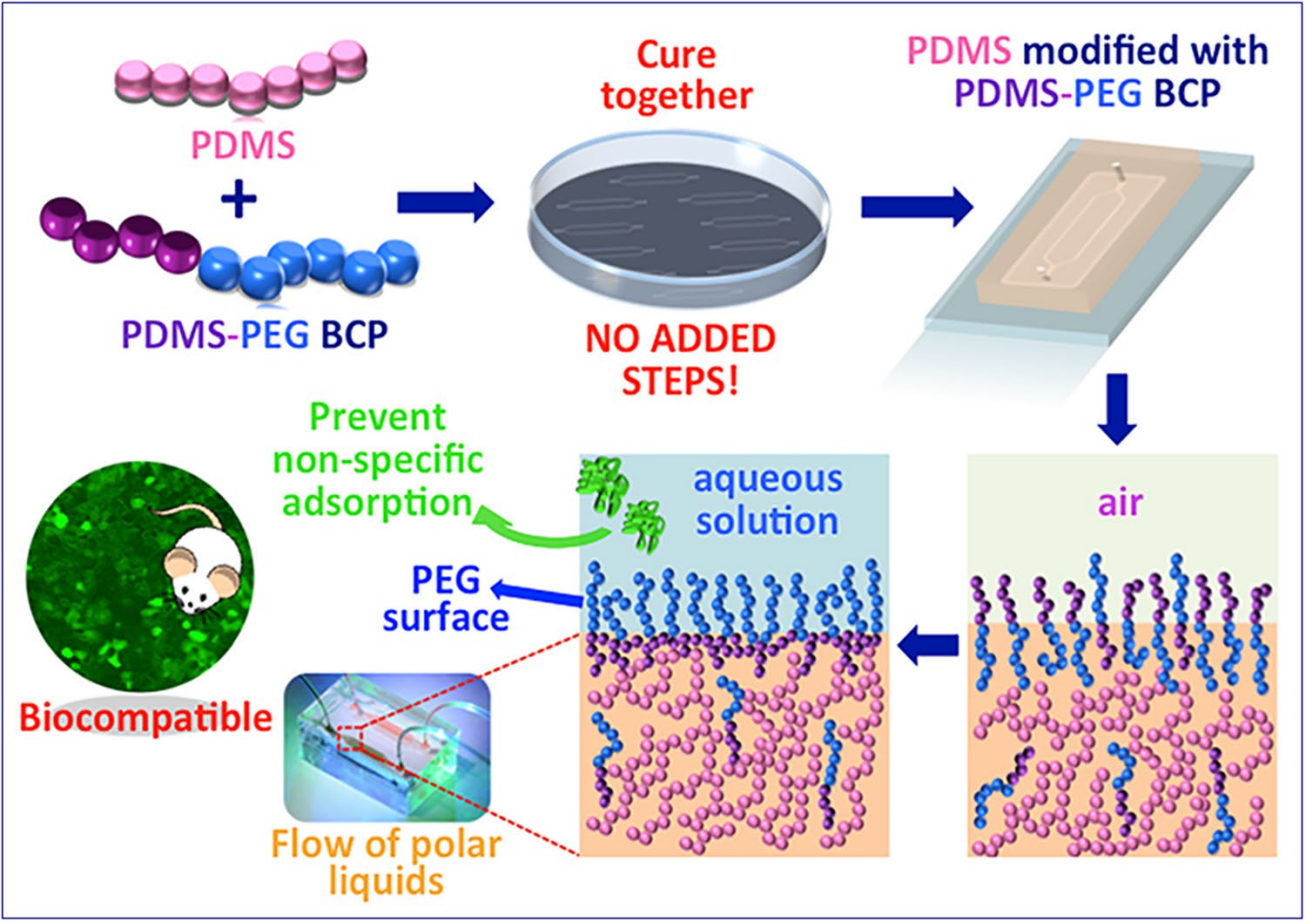
Schematic diagram of the PDMS surface modification method. PDMS and the PDMS-PEG BCP additives are blended, and the device is fabricated following usual processes (no added steps). The copolymers segregate to the PDMS surface in air. When in contact with water, surface rearrangement creates a surface covered with PEG groups that prevent non-specific adsorption of proteins and allows the flow of polar liquids.

We selected a PDMS-PEG BCP as the smart copolymer additive for hydrophilizing the PDMS surface. This copolymer, a commercially available surfactant (Gelest, product code DBE-712), includes a hydrophobic PDMS segment compatible with the base elastomer (e.g. PDMS) and a hydrophilic, fouling resistant PEG block. Its molar mass is 600, and contains 60–70% PEG. The PDMS segment solubilizes the additive within the elastomer matrix during preparation and then anchors the additive in the cured PDMS. It can also be linked with the base PDMS during the plasma treatment used for bonding the device together, improving the longevity of the surface modification. The short chain length and BCP architecture of the additive leads to its segregation to the sample surface^18,27^. When the sample surface is exposed to water (e.g. when the microfluidic channel is filled with aqueous media), the copolymer self-organizes at the PDMS/water interface to expose the PEG segments to the aqueous solution and create a stable hydrophilic surface that prevents the adsorption of proteins and other bio-macromolecules (Fig. 1) without using any additional steps or changing the manufacturing process.

To date, most approaches, to reduce hydrophobicity of PDMS, were developed using post-treatment methods^8,23,28–31^ that add several new, cumbersome steps to the micro-device manufacturing process, often requiring special equipment and/or hazardous routes^9^. This renders them unfavorable for large-scale fabrication and for adoption by a wide user base. Further, they cannot be adapted to the manufacture of other silicone-based elastomers (e.g. tubing, seals). This limits their impact. The approach we present here is differentiated by its simplicity during use, compared with other approaches that rely on coatings or post-processing (Table 1).

While a handful of studies have utilized surface-segregating amphiphilic copolymers to improve the surface hydrophilicity of PDMS, none have demonstrated high degrees of hydrophilicity without loss in mechanical properties and/or optical clarity (Table 1). Furthermore, these studies have almost exclusively focused on characterizing surfaces that have not been subjected to the full slew of processes involved in microfluidic device manufacture, including an alcohol soak for disinfection and plasma treatment for bonding of the device. These processes can leach these additives and/or significantly alter surface chemistry. In addition, none of the past studies characterize the viability of cells upon exposure to these PDMS blends. These amphiphilic additives may leach into the feed going through a biomicrofluidic device, killing cells and thus rendering these approaches moot in practical settings. Thus, there is a significant knowledge gap in not only developing novel additives for PDMS for surface modification but also in better understanding their behavior throughout the life cycle of a biomicrofluidic device.

### Hydrophilicity and wettability of PDMS with PDMS-PEG BCP additives

**Figure 2:**
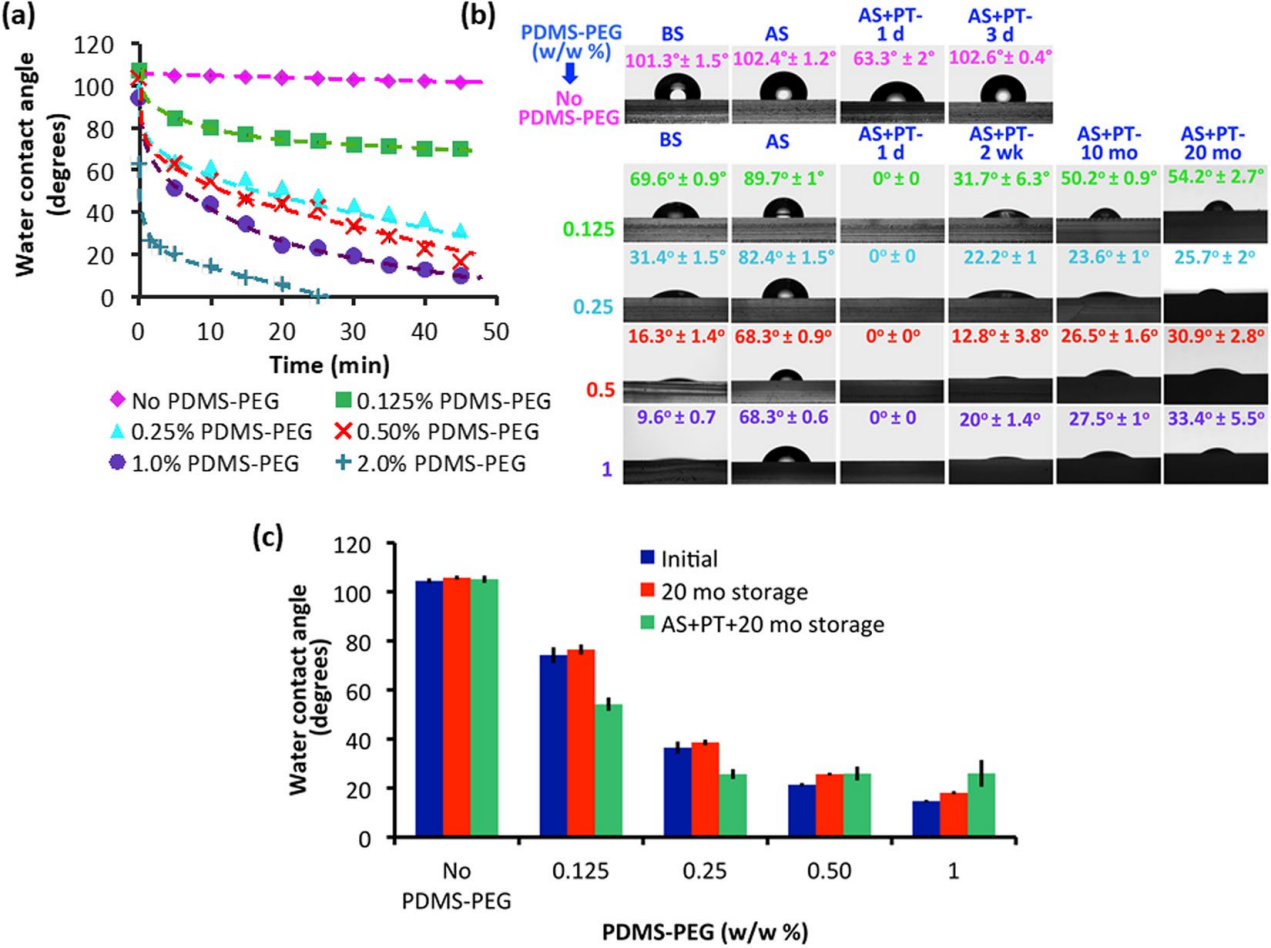
PDMS with PDMS-PEG BCP additives dramatically reduces hydrophobicity. (**a**) The change in WCA with time for various additive ratios (0.125–2%), showing polymer reorganization. (**b**) WCA of PDMS with/without PDMS-PEG BCP additives after soaking them IPA for 24 hours (AS) and treating them with O_2_ plasma (AS + PT) at different time intervals. (**c**) Final (t = 45 min) WCAs for different PDMS-PEG BCP additive ratios after 20 months of storage (with/without plasma treatment) show the stability of the modified materials, indicating that the samples prepared with PDMS-PEG BCP additives do not lose their hydrophilic characteristics for a long period. The data are shown as the mean ± SD (n = 3).

To test our hypothesis that the PDMS-PEG BCP additive would lead to increased hydrophilicity that remains stable over long timescales, we measured sessile drop water contact angles (WCA) on PDMS-PEG BCP modified PDMS surfaces and compared them to the unmodified PDMS over a 20-month duration. We used dynamic contact angle measurements, which are useful for evaluating the wettability and hydrophilicity of modified PDMS surfaces^32,33^. Figure 2a shows the variation of the WCA of PDMS samples prepared with varying amounts of PDMS-PEG BCP additive in time. The initial contact angles of all samples (except the one containing 2% PDMS-PEG BCP additive) were quite high, between 94–106°. This indicates that in air, the sample surface is mostly covered with hydrophobic PDMS segments. However, while the WCA of PDMS with no PDMS-PEG BCP remained steady above 101° during the 45-minute experiments, the WCA of all PDMS with PDMS-PEG BCP additives decreased in time. Furthermore, this decrease was generally proportional to the concentration of PDMS-PEG BCP additives. After 45 minutes of exposure to water, the PDMS-PEG BCP additive containing sample surfaces became significantly more hydrophilic than additive-free PDMS. As little as 0.125% PDMS-PEG BCP additive led to a final contact angle of 69.6° (Supporting Information Fig. S1), comparable with the lowest contact angles reported for other additive-modified PDMS systems^20–22^. The highest BCP containing samples (1.5% and 2% PDMS-PEG BCP) were fully wetted (WCA ≈ 0°) in our dynamic measurements. Nevertheless, it is important to note that increasing BCP concentration for reducing hydrophobicity is not the only requirement for successful and stable surface modification. We encountered bonding problems on glass slides during oxygen plasma treatment at higher copolymer concentrations (1.5 and 2 (w/w %)), so we eliminated these concentrations for further experiments.

These results confirm that upon exposure to water, the PDMS-PEG BCP additive self-assembles at the interface to create a hydrophilic PEG layer, and indicate that this rearrangement occurs faster and more effectively with increasing PDMS-PEG BCP content. Furthermore, this approach can lead to final WCA values much below previous reports for additive-modified PDMS materials, which range between 84° and 63°^20,21^.

In practical applications, PDMS is not used directly after molding. PDMS devices are typically sterilized by immersion into an alcohol such as IPA, which may leach out additives. Then, they are treated with O_2_ plasma and bonded to glass, a silicon wafer, or another piece of PDMS. It is critical for the improved surface hydrophilicity to be stable during these processing steps. Furthermore, microfluidic devices are not necessarily used directly after manufacture. Therefore, the modified surface needs to be stable over long time periods.

To test these parameters, we first established the soaking time of PDMS with and without PDMS-PEG BCP additives in IPA. We measured the WCA of PDMS without PDMS-PEG and of PDMS with 0.5% PDMS-PEG BCP additive after soaking in IPA for 6, 12 and 24 hours (Supporting Information Fig. S2). The hydrophilicity of samples after 6 hours of IPA soaking was higher compared with that of samples with 12 hours and 24 hours of soaking. We believe that during soaking in IPA, the lower molar mass fractions of the PDMS-PEG BCP diffused out^21^. This resulted in a decrease in copolymer concentration in the PDMS and a significant increase in the contact angle. We observed that 12 hours of IPA soaking was sufficient to remove all the lower mass fractions of the BCP because no significant change in hydrophilicity was observed between 12 hours and 24 hours IPA soaking. Still, some PDMS-PEG BCP remained in the PDMS as the final contact angles were still much lower than that of PDMS with no PDMS-PEG BCP. The remaining PDMS-PEG BCP molecules were likely of higher molar mass, which improved the long-term stability of the layer. Furthermore, this decreased the risk of the additive, particularly low molar mass fractions likely to act as cytotoxic surfactants, leaching out of the PDMS during operation, which could negatively impact cell viability. Nonetheless, we selected 24 h IPA soaking for experimental practicality and to ensure consistent results in further experiments (samples labeled AS).

The improved surface hydrophilicity of IPA-soaked samples was stable for at least 20 months (Fig. S3). Some of the samples were then treated with O_2_ plasma (samples labeled AS + PT) (Fig. 2b). A day after O_2_ plasma treatment (AS + PT 1 d), the hydrophobicities of PDMS samples both with and without PDMS-PEG BCP additives were significantly reduced (Fig. 2b). The WCA of PDMS with no PDMS-PEG BCP became 63.3°.

It has previously been reported that PDMS surfaces exposed to plasma exhibit increased oxygen content and possibly silicon (Si) atoms are bonded to three or four oxygen atoms and that this reduces hyrophobicity^34^. The main challenge posed by the plasma oxidation is the eventual hydrophobic recovery. This is a result of the reorientation of pre-existing oligomers from the bulk to the surface^35^. Indeed, 3 days after plasma treatment (AS + PT 3 d), the WCA of the PDMS with no PDMS-PEG BCP additive returned to its initial value of 102°. PDMS with PDMS-PEG BCP additives also exhibited an increase in hydrophilicity upon O_2_ plasma treatment. The surfaces became fully wettable a day after plasma treatment with WCA values around 0°. As seen in Fig. 2b, although a minor increase in wettability was observed, all PDMS with PDMS-PEG BCP additives maintained their hydrophilicity, with WCA values between 54.2° ± 2.7° and 25.7° ± 2°. These values are significantly lower than previous reports^20–22^. We believe that the existence of PEG on the modified PDMS surface enhances Si-O bonding and as a result, more SiO_x_-rich layer and more hydrophilic surfaces can be obtained as compared to PDMS with no PDMS-PEG BCP. Importantly, this enhanced surface hydrophilicity was stable for at least twenty months. The increased surface hydrophilicity may have enhanced the surface segregation of the PDMS-PEG BCP by creating a local gradient, drawing the copolymer to the surface even before exposure to water. The increased degree and stability of surface hydrophilicity may also be linked with the complex and competing etching, deposition and reaction processes that occur during plasma treatment. During the O_2_ plasma treatment, PDMS repeat units are partially etched on the surface, losing their methyl groups and forming silica. The plasma treatment may also cause cross-linking, but this effect is relatively limited in PDMS^36^. In contrast, oxygen-containing polymers such as PEO tend to undergo atomic re-arrangement reactions such as cross-linking as opposed to etching^37^. This implies that the plasma treatment may preferentially etch the hydrophobic methyl groups from PDMS chains on the surface, exposing PEG segments that were right below. The plasma treatment can also chemically cross-link the PDMS-PEG BCP additive to the PDMS network. Furthermore, it may lead to cross-linking between PEO chains on the surface. This may anchor the PDMS-PEG BCP specifically on the top surface of the sample, improving the longevity of surface modification. Additionally, PDMS samples with different PDMS-PEG BCP preserved their hydrophilic characteristics even after 20 months of storage (with/without plasma treatment) indicating that samples prepared with PDMS-PEG BCP additives are stable for a long period (Fig. 2c).

### Characterization of the physical properties of PDMS with PDMS-PEG BCP additives

#### Transparency

Microfluidic devices are commonly used together with bright field and fluorescence microscopy for imaging cells^38^ to monitor their health and motility. Therefore, materials used for manufacturing such devices must be transparent. Blue light [460–500 nm] excitation is commonly used to image green fluorescent protein (GFP) and Calcein AM, and green light [528–553 nm] excitation is useful for imaging red fluorophores^38^.

Accordingly, we assessed the optical clarity of PDMS with and without PDMS-PEG BCP additives by measuring light transmittance through 8 mm thick slabs between 400–600 nm wavelengths in the UV-visible range before and after an IPA soak (Supporting Information Fig. S4) after the fabrication. Transmittance values for the center wavelengths of blue light (480 nm) and green light (540 nm) are given in Table 2. Before soaking in IPA (Fig. S4(a)), transparency values for PDMS with up to 0.5% PDMS-PEG BCP were comparable to additive free PDMS, with all transmittance values above 96%. The transparency of the PDMS sample with 1% PDMS-PEG was slightly lower, with transmittance values in the 80–88% range. This may arise from the formation of micelles or similar aggregates of the PDMS-PEG BCP surfactant within the bulk PDMS at these higher concentrations, as observed in other studies^20,21^. After soaking in IPA (Fig. S4(b)), samples modified with 0.125% and 0.25% PDMS-PEG BCP additives exhibited approximately the same optical clarity as unmodified PDMS. However, the optical clarity of modified PDMS with 0.5% and 1% PDMS-PEG BCP decreased, with transmittance values around 75% and 50%, respectively. PDMS samples containing 0.25% PDMS-PEG BCP successfully combined high optical clarity with a hydrophilic surface.

**Table 2:** Optical and mechanical properties of PDMS with PDMS-PEG BCP additives.

(PDMS-PEG) (w/w %)	Transmittance (%) BS/AS (480 nm)	Transmittance (%) BS/AS (540 nm)	Young’s Modulus BS/20 mo (MPa)	Compressive Modulus BS/20 mo (MPa)
No PDMS-PEG^a^	100 ± 0.03/100 ± 0.02	100 ± 0.02/100 ± 0.03	1.3 ± 0.10	187 ± 5
No PDMS-PEG	100 ± 0.01/100 ± 0.01	100 ± 0.02/100 ± 0.02	1.2 ± 0.10/1.2 ± 0.2	217 ± 3/212 ± 5
0.125	100 ± 0.02/100 ± 0.01	100 ± 0.01/100 ± 0.01	1.3 ± 0.03/1.2 ± 0.1	218 ± 3/221 ± 3
0.25	100 ± 0.02/99 ± 0.01	100 ± 0.02/99 ± 0.02	1.4 ± 0.02/1.2 ± 0.2	203 ± 5/213 ± 7
0.5	97 ± 0.01/78 ± 0.01	98 ± 0.01/76 ± 0.01	1.3 ± 0.02/1.3 ± 0.05	201 ± 8/210 ± 3
1.0	84 ± 0.04/57 ± 0.03	87 ± 0.03/55 ± 0.04	1.2 ± 0.10/1.3 ± 0.2	219 ± 1/225 ± 3

^a^Young’s modulus and compressive modulus of PDMS with no PDMS-PEG BPC from literature^40^.

BS: Before IPA Soaking, AS: After IPA Soaking, 20 mo: 20 mo storage without any IPA soak or O_2_ plasma treatment. The data are shown as the mean ± SD (n = 3).

#### Surface Characterization

**Figure 3:**
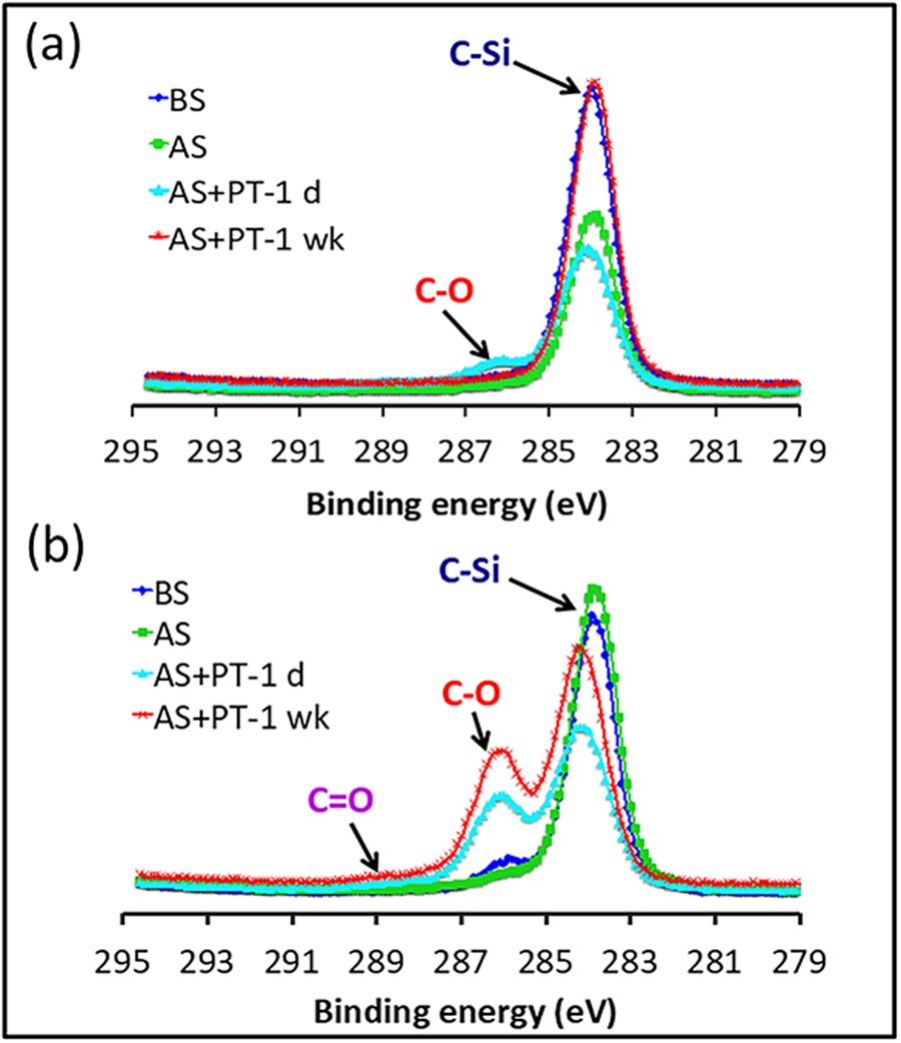
High-resolution scans of C1s of both (**a**) PDMS with no PDMS-PEG BCP additive and (**b**) PDMS with 0.25% PDMS-PEG BCP additive before IPA soaking (BS), after IPA soaking (AS), after IPA soaking and 1 day after O_2_ plasma treatment (AS + PT-1 d) and after IPA soaking and 1 week after O_2_ plasma treatment (AS + PT-1 wk) were analyzed. Unmodified PDMS acquires hydrophilic groups upon plasma treatment, but these functional groups disappear from the surface within a week. XPS data confirms that the PDMS-PEG BCP additive segregates to the surface, and creates hydrophilic groups on the surface upon plasma treatment.

We used X-ray photoelectron spectroscopy (XPS) to gain a better understanding of the changes in surface chemistry during the manufacture of biomicrofluidic devices from PDMS with and without the PDMS-PEG BCP additive. In this study, we focused on PDMS with 0.25% PDMS-PEG BCP, selected according to the criteria described above, and PDMS with no PDMS-PEG BCP. We analyzed their surface chemistry at each stage of the microfluidic device manufacture process. The elemental surface compositions of both the PDMS with no PDMS-PEG and 0.25% PDMS-PEG, determined by the survey scan, remained essentially unchanged after soaking in IPA (Supporting Information Fig. S5). After plasma treatment, survey scans of PDMS with and without PDMS-PEG BCP additives both indicate an increase in carbon and oxygen content and a corresponding decrease in silicon content (Supporting Information Fig. S6). High-resolution scans of C1s spectra were used to gain deeper insight into the chemical changes that occurred during these processes (Fig. 3(a,b)).

PDMS with no PDMS-PEG spectra featured only one peak, at 284.2 eV, corresponding to C-Si bonds. This was unchanged upon soaking in IPA. Upon plasma treatment, peaks appeared at 286.3 eV and 289.1 eV, assigned to C-O and C=O bonds, respectively^39^. These peaks, however, completely disappeared after a week. This is due to the recovery of hydrophobicity after oxidation by reorientation of the surface silanol groups into the bulk polymer, which provides for the movement of free PDMS chains from the bulk phase to the surface and condensation of silanol groups at the surface^8^. In contrast, PDMS with 0.25% PDMS-PEG BCP showed both a strong peak near 284.6 eV arising from C-Si bonds and a shoulder near 286.3 eV, corresponding to C-O bonds, indicating the presence of PEG segments from the PDMS-PEG BCP additive near the surface. Upon plasma treatment, the intensity of the C-O peak increased, and a new peak corresponding to C=O appeared. These groups may arise both from reactions of PDMS and from reactions and cross-linking of PEG. Unlike pure PDMS, the intensity of the C-O and C=O peaks in the PDMS with 0.25% PDMS-PEG remained unchanged a week after plasma treatment. This phenomenon confirms the existence of PEG molecules on the modified surface for long-term stability after plasma treatment, which is in good agreement with the hydrophilicity data (Fig. 2a,b).

#### Mechanical properties

PDMS is a good candidate for use in microfluidic devices due to its high compliance and flexibility. Its Young’s modulus depends on the exact formulation, and is around ∼1.32–2.12 MPa for the commonly used pre-polymer to curing agent ratio of 10:1^40–42^. Ideally, surface modification by any approach should not compromise these mechanical properties. To check the mechanical properties of PDMS-PEG-modified PDMS samples, tensile strength and compressive modulus were evaluated by dynamic mechanical analysis (DMA) immediately after fabrication and also 20 months after fabrication. Young’s modulus and compressive modulus of the modified samples were calculated for the linear elastic region (<40% strain). No significant change was observed with the mechanical properties of the PDMS-PEG BCP modified PDMS when compared with literature studies even after 20 months of storage.

### Biocompatibility

Many microfluidic applications that utilize PDMS and its alternatives involve culture or circulation of cells from different tissues. Therefore, when designing a new material for biomicrofluidics, it is crucial to take its biocompatibility into account. For instance, the surface modifying additives may leach from the device into the microfluidic channel and affect cell viability and/or function. This may lead to poor device performance even if surface hydrophilicity is enhanced. To date, there are some studies that evaluated the biocompatibility or cell adhesion of modified PDMS microfluidic devices or slabs using mammalian A549 cells^43^, L929 mouse fibroblasts^44^, tendon stem cells^45^, mesenchymal stem cells (MSCs)^46^, brain cerebral cortex cells^47^, HeLa cells^48^, and stroma cells^49^. To our knowledge, none of the previous PDMS modification strategies were evaluated for compatibility with hepatocytes, the parenchymal cells of the liver, which are highly susceptible to adverse reactions. The liver plays a central role in drug metabolism and detoxification so the development of liver-on-a-chip models for successful prediction of toxic response is at the center of the recent initiatives towards *in vitro* human clinical trial approaches^50,51^. Here, we used rat primary hepatocytes to test the biocompatibility of our modified PDMS substrate in a simple microfluidic liver-on-a-chip model.

**Figure 4:**
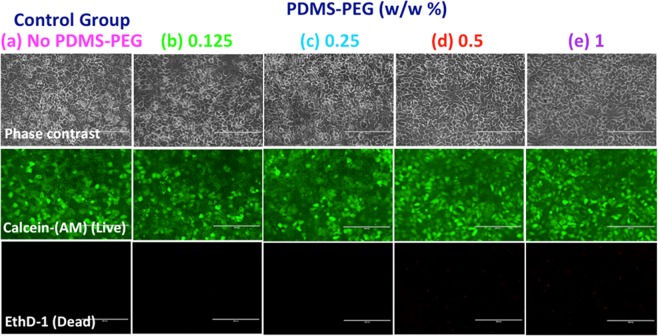
Biocompatibility of PDMS with PDMS-PEG BCP additives. Rat hepatocytes were cultured in glass-(modified) PDMS devices. No adverse effects were observed (3 days) with (**a**) PDMS with no PDMS-PEG and PDMS with (**b**) 0.125%, (**c**) 0.25%, (**d**) 0.5% and (**e**) 1% (w/w) PDMS-PEG BCP. Image scale bar: 400 μm. Each experiment was conducted in triplicates from at least three different rat isolations.

To ensure that the use of the PDMS-PEG BCP in microfluidic device manufacture does not adversely impact cell function, we manufactured microfluidic devices using a glass bottom and PDMS top with or without PDMS-PEG BCP additives, and cultured primary rat hepatocytes in these devices. In order to quantitatively evaluate cell viability, the cells were stained with a live (green)/dead (red) stain 3 days after the culture (Fig. 4). Cells had high viability (>99.0%) throughout the 3 day culture period following the initial cell seeding into the microdevice. The use of the PDMS-PEG BCP additive led to no visible or significant differences in cell viability or morphology. PDMS-PEG modified microfluidic devices performed just as well as PDMS with no PDMS-PEG additives and presented no adverse effects. Since *in vitro* systems are often preferred as models to predict drug toxicity and pharmacokinetics for clinical cases, this design can be easily scaled to create an array of *in vitro* studies for rapid drug development or studying the toxicity of drugs due to the simplicity of the device.

### Protein adsorption on PDMS with PDMS-PEG BCP additives

**Figure 5:**
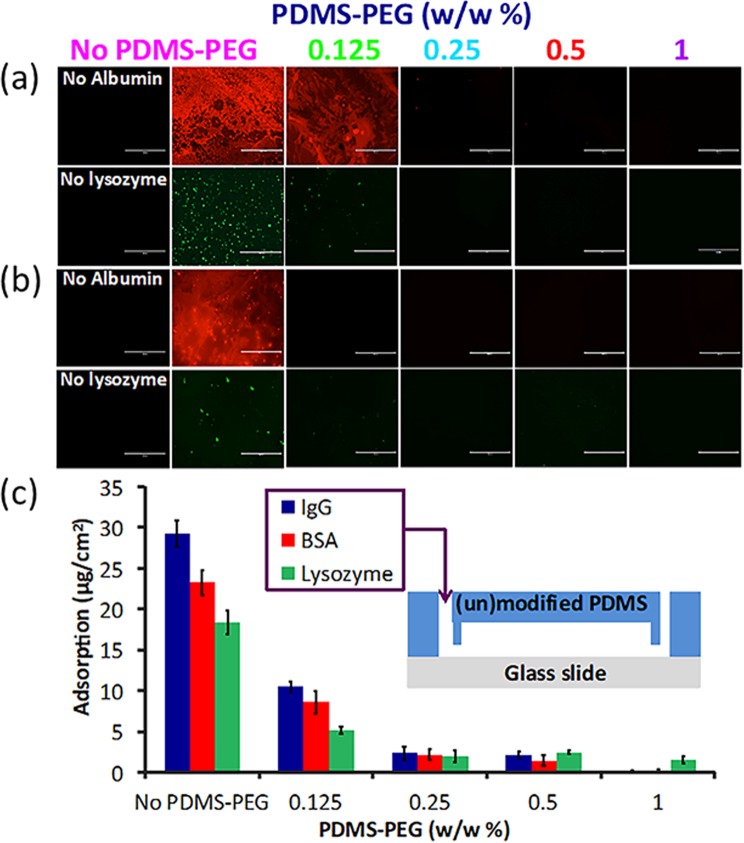
PDMS with PDMS-PEG BCP additives exhibit lower protein adsorption. Adsorption of fluorescently labeled albumin and lysozyme onto BCP modified PDMS slabs (**a**) without any treatment (**b**) after IPA soak and 1 week after O_2_ plasma. Samples were covered with protein solutions for 30–90 minutes. PDMS with PDMS-PEG BCP additive showed high adsorption, whereas PDMS modified with 1% w/w PDMS-PEG block copolymer exhibited significantly lower adsorption, near detection limit (n = 5). Image scale bar: 400 μm. (**c**) We measured adsorption of IgG, BSA, and lysozyme in modified microfluidic devices, comparing the influx and efflux concentrations after IPA soaking and a week after O_2_ plasma. PDMS-PEG BCP additive leads to significantly reduced adsorption for all proteins. Error bars represent the standard deviation with samples measured in triplicate (n = 3).

The main goal of developing this PDMS surface modification approach was to create a fouling resistant surface and prevent the non-specific adsorption of proteins onto the microfluidic device. This is motivated by two phenomena. First, most of the undesired bioreactions and bio-responses in artificial materials are promoted due to adsorbed proteins^52,53^. Second, many applications of biomicrofluidics involve controlling the exposure of cells to a known concentration of a specific, desired protein such as a biologic drug. Non-specific adsorption leads to the loss of this drug through adsorption, exposing the cells to a lower concentration than presumed. This can lead to a severe underestimation of the toxicity and activity of such drugs. While hydrophilicity is broadly correlated with decreased protein adsorption, the relationship is not necessarily straightforward^54,55^. Therefore, we quantitatively measured the adsorption of two fluorescently-labeled proteins, albumin and lysozyme, on PDMS slabs with and without PDMS-PEG BCP additives (Fig. 5(a,b)), both directly upon manufacture (Fig. 5a) and following processes that simulate biomicrofluidic device manufacture (Fig. 5b, IPA soak and 1 week after O_2_ plasma treatment).

PDMS with no PDMS-PEG BCP adsorbed significantly more protein than all PDMS with PDMS-PEG-BCP additives, confirming that this approach led to decreased non-specific adsorption (Fig. 5a). PDMS with 0.125% PDMS-PEG BCP exhibited some protein adsorption. No adsorption was visible for any of the other samples. The same trend continued following soaking in IPA and O_2_ plasma treatment (Fig. 5b). PDMS slabs with PDMS-PEG BCP additives indicated substantially reduced adsorption as compared to additive free PDMS.

To further quantify protein adsorption in a more realistic setting for biomicrofluidic device applications, we manufactured microfluidic devices from PDMS with or without PDMS-PEG additives. We then introduced a protein solution containing 0.05 mg/mL BSA, lysozyme or IgG into the microchannel (30–90 min), and measured the loss of protein due to adsorption on the device by micro-BCA analysis (Fig. 5c). Devices with PDMS-PEG additives adsorbed significantly lower quantities of each protein as compared to PDMS with no PDMS-PEG (Fig. 5c). As the BCP concentration increased in the mixture, the amount of adsorbed protein decreased. PDMS-PEG BCP additives significantly reduced protein adsorption at concentrations as low as 0.125% (w/w). The use of only 1% PDMS-PEG additive led to 98.9%, 89.4%, and 99.6% lower adsorption of albumin, lysozyme, and IgG, respectively when compared to PDMS without PDMS-PEG BCP. An additive concentration of 0.25% PDMS-PEG led to a ~90% the reduction in protein adsorption while also retaining excellent optical clarity.

### Capillary-driven microfluidic devices with PDMS-PEG BCP additives

Having demonstrated the successful hydrophilization of PDMS with PDMS-PEG BCP additives, we investigated the flow characteristic of PDMS with and without PDMS-PEG BCP additives (0.25% and 0.5%) in the capillary microchannels which were bonded on the glass substrates. Two linear channels (height: 0.1 mm, length: 40 mm) with different widths (0.25 mm and 0.5 mm) were tested for capillary-driven flow experiments. PDMS with no PDMS-PEG BCP was utilized as a control. All samples were tested 3 days after plasma treatment. Liquid was introduced into the inlet of the capillary channel and fluid flow through the channel was recorded by a camera to calculate the experimental flow rates. Table 3 and Fig. S7 show the variation of flow velocities of liquid using PDMS samples with varying amounts of PDMS-PEG BCP. All modified devices were shown to fill through steady capillary action while PDMS without PDMS-PEG BCP failed to fill with liquid. We did not observe a significant difference in capillary flow rates through the 0.25% and 0.5% PDMS-PEG BCP modified samples. This was consistent with the WCA results, which showed very similar hydrophilicity (Fig. 2). The advantage of the fabrication technique presented here is that hydrophilic PDMS microfluidic channels can be obtained with a simple, one-step method through inexpensive bench-top methods.

**Table 3:** Capillary-driven flow of hydrophilic PDMS channels with aqueous solutions.

PDMS-PEG (w/w %)	Flow rate (nL/s)	
	Width: 0.25 mm	Width: 0.50 mm
No PDMS-PEG	No flow	No flow
0.25	69 ± 2.5	181 ± 2
0.50	69 ± 1.5	200 ± 1

The data are shown as the mean ± SD (n = 3).

We finally evaluated our results to choose the most preferred PDMS-PEG BCP concentration for a given application. As the biocompatibility and mechanical properties of all PDMS samples with PDMS-PEG BCP additives are almost identical with PDMS, we compared the different samples for their transparency, WCA after plasma treatment (t = 45 min) and protein (IgG as a sample protein) adsorption data. We selected 0.25% PDMS-PEG BCP concentration (WCA = 25.7° ± 2°, transmittance = 99%, reduction in IgG adsorption relative to PDMS with no additive = 92.2%) to be the best performing composition for applications where optical clarity is of importance, since the transparency of the modified samples decreased down to 73% and below after IPA soaking with BCP concentrations at or above 0.5%.

## Conclusion

This manuscript introduces a simple approach to address non-specific protein adsorption, a key problem encountered in the use of PDMS in biomicrofluidic applications, without making any changes to the existing workflow for manufacturing such devices. This involves simply adding a PDMS-PEG BCP additive to PDMS during device manufacture. This BCP segregates to the surface during device manufacture and rearranges to create a hydrophilic surface upon exposure to aqueous media. As little as 0.25% additive leads to contact angles as low as 31.4° ± 1.5, whereas 2% additive leads to a fully wettable (WCA ≈ 0) surface. Surface hydrophilicity is retained through common processes used in microfluidic device manufacture (e.g. immersion in IPA and plasma treatment), and after prolonged storage at the bench top for at least 20 months. The extent and durability of surface hydrophilicity obtained by this method surpass others reported in the literature^20–23^. Only 0.25% PDMS-PEG additive leads to ~90% reduction in the adsorption of three proteins, whereas 1% additive led to 89–99.6% reduction in protein adsorption, comparable to or better than the highest reductions in protein adsorption in the literature^56^. While we did not test protein adsorption resistance after extensive storage, the long-term stability of surface hydrophilization implies that these devices will likely exhibit reduced non-specific adsorption for long time periods. Furthermore, devices prepared with this approach preserve their transparency, flexibility, and biocompatibility with primary rat hepatocytes. According to all results, 0.25% (w/w) copolymer concentration was selected as an optimum value for applications requiring high transparency, whereas 1% additive led to samples with the lowest fouling while preserving mechanical properties.

The PDMS modification method introduced here does not require any additional steps or equipment for device fabrication. This allows easy adoption and scale-up and is more compatible with mass production of microfluidic devices compared to silicon, glass or thermoplastic alternatives. It is our opinion that this method has a potential for applications including drug-related studies, analytical separations, biosensing, cell targeting, and isolation. Apart from the applications in microfluidics, we expect our invention to remove barriers that currently prevent the use of PDMS in critical commercial applications such as those in applications in pharmaceutical and biomedical industries.

## Methods

### Microfluidic device fabrication for cell culture studies and protein adsorption experiments

Silicon wafer templates served as negative molds to fabricate microfluidic devices using PDMS, (Sylgard 184, Dow Corning, Tewksbury, MA) with and without PDMS-PEG BCP additives and utilizing standard soft lithography protocols. The microfluidic platform consisted of media fluid inlet/outlet and cell inlet/outlet in the same place, and a cell culture chamber. The dimensions of the chamber were 11 mm^2^ × 0.1 mm (Surface area x height). Inlet and outlet ports of the device were punched into the PDMS microfluidic device using a 1.5 mm biopsy punch piercing tool (Ted Pella Inc.). The face of the PDMS with microchannel and a glass microscope slides (75 × 25 mm, Thermo scientific) were bonded with O_2_ plasma (80 W, 35 sec) using a vacuum plasma cleaner.

### Capillary-driven microfluidic device fabrication

Capillary-driven microfluidic devices were fabricated with and without PDMS-PEG BCP using replica molding on silicon wafer templates as discussed above. Two linear microfluidic channel designs consisted of media fluid inlet/outlet were fabricated with varied geometries (0.25 mm, 0.5 mm widths, 0.1 mm height, and 40 mm length). Inlet and outlet ports of the microfluidic devices were punched using a 3.5 mm biopsy punch piercing tool (Ted Pella Inc.). The devices were then bonded to glass microscope slides (75 × 25 mm, Thermo Scientific) using an O_2_ plasma cleaner (80 W, 35 sec). We placed a drop of DDI water with food coloring into the inlet port of the capillary channel with no applied pressure. The progress of the aqueous solution through the capillary was recorded using a camera. The recording was analyzed to calculate the experimental capillary flow rates.

### Production of PDMS with PDMS-PEG BCP additives

A block copolymer with a poly(dimethylsiloxane) (PDMS) and hydrophilic poly(ethylene glycol) (PEG) blocks, PDMS-PEG, was purchased from Gelest (product code DBE-712, dimethylsiloxane-(60–70% ethylene oxide) block copolymer, MW 600, 20 cSt, specific gravity: 1.01, refractive index: 1.442, Gelest, USA) and utilized as an additive in the modification of microfluidic devices. Silicone pre-polymer and curing agent were mixed in a mass ratio of 10:1 (w/w). Desired amount of PDMS-PEG BCP was then added to the polymer base-curing agent mix to obtain a final additive concentration of 0.125%, 0.25%, 0.5% 1.0%, 1.5%, 2.0% (w/w) in the mixtures. The mixtures were blended using a glass a rod and poured onto a silicon wafer or into a petri dish for the fabrication of microfluidic devices and slabs, respectively. Trapped air bubbles were removed by keeping the mixture at +4 °C for 15 min. After removing air bubbles, the blended mixture was cured at 70 °C for 24 h. All devices and slabs (~2 mm thick) were rinsed with isopropyl alcohol (IPA) for 24 h and dried at room temperature (RT). Steam sterilization was applied to microfluidic devices before performing experiments.

### Primary rat hepatocyte isolation and cell seeding

Primary rat hepatocytes were isolated from adult female Lewis rats (Charles River Laboratories, MA) as described previously^57^. All methods were performed in accordance with the guidelines and regulations of National Research Council. For isolation, protocol #2011N000111 approved by the Institutional Animal Care and Use Committee (IACUC) at the Massachusetts General Hospital (MGH) was implemented by the Cell Resource Core (CRC). In general, as determined by trypan blue exclusion, 100–150 million hepatocytes with 90–95% cell viability were obtained and a suspension consisting of primary rat hepatocytes at a final concentration of 5 million cells (M) mL^−1^ was prepared to plate into microfluidic devices. Before introducing rat hepatocytes, glass bottom of the devices was coated with 50 μg/mL fibronectin (Sigma-Aldrich) for 30–45 min at 37 °C in 5% CO_2_. Then the cells were plated into the cell culture chamber and the device was connected to a syringe pump with a flow rate of 10 μl/hr and incubated at 37 °C in 5% CO_2_. After 24 hours of seeding, the flow of the fresh media was replaced in the cell culture chamber of perfusion devices and continued thereafter. Dulbecco’s modified eagle’s medium (DMEM, Life Technologies, Carlsbad, CA, USA) supplemented with 10% fetal bovine serum (FBS, Sigma, St. Louis, MO, USA), 0.5 U/mL insulin, 7 ng/mL glucagon, 20 ng/mL epidermal growth factor, 7.5 μg/mL hydrocortisone, 200 U/mL penicillin, 200 μg/mL streptomycin, and 50 μg/mL gentamycin was utilized for culturing primary rat hepatocytes. For all fluidic connections and media perfusion, Tygon tubing (0.01“ID × 0.03” OD, Cole Parmer) was used.

### Hepatocyte morphology and cell viability

Hepatocyte morphology and viability were assessed by phase contrast microscopy (Evos FL Imaging System, ThermoFisher Scientific). Live/Dead Cell Viability/Cytotoxicity Kit (Thermo Fisher Scientific) were utilized to determine cell viability. For this purpose, Live/Dead assay reagents (calcein AM (10 μL), ethidium homodimer-1 (100 μL)) and PBS (2.5 mL) were combined and vortexed to ensure thorough mixing. Reagents were introduced into the culture chamber and after 30 min incubation (37 °C) and PBS rinsing, images were captured on the EVOS fluorescence microscope to evaluate the cell viability.

### Protein adsorption study

PDMS-PEG BCP at ratios between 0.125–1.0 (w/w %) was blended with PDMS and poured into a petri dish and cured at 70 °C for 24 h, as described in Section 2.2. After polymerization, round swatches of PDMS samples (5 mm Dia × 4 mm) were cut using a 5 mm dermal punch (Ted Pella Inc.). These samples were immersed in phosphate buffered saline (PBS, pH 7.4) for 2 h to reach pre-equilibration. 0.5 mg/mL solutions of each fluorescently labeled protein, bovine serum albumin (BSA) (Alexa Fluor 594-labeled BSA, Thermo Fisher Scientific) or lysozyme (FITC-labeled, Nanocs), were prepared in PBS separately. To study protein adsorption, 50 μL of fluorescently labeled protein solution was placed on the modified PDMS swatch and incubated in the dark at 37 °C for 1.5 h. For comparison, the same procedure was followed for PDMS with no PDMS-PEG BCP. After 1.5 h, each sample was rinsed with 200 μL PBS. Fluorescence microscope images were captured by Evos FL Imaging System (ThermoFisher Scientific) using 10X objective. Quantitative protein adsorption experiments were also performed using PDMS microfluidic devices with/without PDMS-PEG additives. For this purpose, microfluidic devices were conditioned with PBS at a flow rate of 20 μL/min for 4 hours using a syringe pump and then emptied. 0.05 mg/mL solutions of BSA from the chicken egg (Sigma Aldrich), lysozyme from chicken egg white (Sigma Aldrich) and Immunoglobulin G from human serum (IgG) (Sigma Aldrich) were introduced into the device (30–90 min). The amount of adsorbed BSA, lysozyme and IgG were measured comparing the influx and efflux concentrations utilizing the Pierce BCA Protein Assay Kit (Thermo Scientific) according to the manufacturer’s protocol.

### Characterization

#### Optical Properties

Optical clarity was quantified using UV-Vis spectrophotometer (Thermo Scientific, Genesys 10S equipped with a high-intensity xenon lamp and dual-beam optical geometry) within the wavelength range of 400–600 nm both for PDMS and BCP modified PDMS samples (0.125–1.0% (w/w)). Samples were tested before and after IPA soaking. All samples were prepared with similar thicknesses (~8 mm) with the purpose of avoiding any disparity in the data.

#### Mechanical properties

Mechanical properties (Young’s modulus, compressive modulus) were tested using TA Instruments RSAIII Dynamic Mechanical Analyzer (DMA), (Rheometrics Solids Analyzer). PDMS samples with/without PDMS-PEG additives for tensile and compressive testing were fabricated according to ASTM standards. For tensile testing, crosshead velocity was 250 mm/min. At strain levels below 40%, the linear behavior allows utilizing Hooke’s law (E = σ/ε, where σ is the applied stress and ε is the resultant strain) to calculate Young’s modulus^40^. For compression testing, crosshead velocity was set to a maximum of 20 mm/min. Prior to all subsequent compression tests, a drop of machine oil was applied to the parallel surfaces of the PDMS cylinder to prevent excessive friction and the resultant barreling.

#### Surface characterization

Sessile drop water droplet contact angles (WCA) were measured at the polymer-air interface using a contact angle goniometer (Rame-Hart Instrument Co., Netcong, NJ) to assess the wettability of PDMS modified with PDMS-PEG BCP additives. Briefly, 6 μL volume of distilled water (18.2 MΩ cm^−1^ water) was dropped onto the BCP modified PDMS slab (2 cm × 2 cm) and the contact angle was measured at regular time intervals to observe the timeline of surface arrangement. WCA of PDMS without BCP additive substrates (2 cm × 2 cm) was also measured as a control.

To characterize the surface chemistry of PDMS with and without PDMS-PEG BCP additives, square samples (1 cm × 1 cm) were prepared. Samples were analyzed using X-ray photoelectron spectroscopy (XPS) using the K-Alpha + XPS system (Thermo Scientific) at Harvard University’s Center for Nanoscale Systems. The probe for the measurement was aluminum k-α X-ray line with energy at 1.4866 keV and X-ray spot size at 400 μm with 90 degrees take-off angle (sampling depth is around 10 nm from the surface). A flood gun, which supplies low energy electrons and ions was used throughout the entire experiment for sample surface charge compensation. Both survey spectra and high-resolution scan data were collected at each sample. For survey spectra, the scan was completed by taking an average of 5 scans in 1 eV steps with passing energy at 200 eV from −10 eV to 1350 eV binding energy. For high-resolution scans, the data were collected by taking an average of 10 scans in 0.1 eV steps with passing energy at 50 eV for Si 2p, O 1s, and C 1s photoelectron lines.

#### Statistical analysis

Each biocompatibility experiment was conducted in triplicate using cells from at least three different rat isolations. Three different samples were utilized to quantify the WCA, protein adsorption, and mechanical analysis measurements. Capillary-driven flow experiments were performed with three different samples (n = 3). XPS of each sample was obtained by taking an average of 5 and 10 scans for survey spectrum and high resolution scan data respectively. Wherever indicated, quantitative data were plotted as the mean ± standard error of the mean (n = 3).

## Supplementary information

Supporting Information

## Data Availability

The raw and processed data to reproduce these findings can be obtained by contacting the authors.
