# Supplementary material for: Simple Surface Modification of Poly(dimethylsiloxane) via Surface Segregating Smart Polymers for Biomicrofluidics
Source: Sci Rep. 2019 May 14;9:7377. doi: 10.1038/s41598-019-43625-5 (PMC6517421; doi:10.1038/s41598-019-43625-5)
Supplement: Supplementary file 1 — Supporting Information [file 41598_2019_43625_MOESM1_ESM.pdf]

Supporting Information

Simple Surface Modification of Poly(dimethylsiloxane) via Surface Segregating Smart Polymers for Biomicrofluidics

Aslıhan Gökaltun[1,2,3], Young Bok (Abraham) Kang[1], Martin L. Yarmush[1,4], O. Berk Usta[1], Ayşe Asatekin[2*]*

1. Center for Engineering in Medicine at Massachusetts General Hospital, Harvard Medical School, and Shriners Hospital for Children, 51 Blossom St., Boston, MA, 02114

2- Department of Chemical and Biological Engineering, Tufts University, 4 Colby Street, Medford, MA 02474

3- Department of Chemical Engineering, Hacettepe University, 06532, Beytepe, Ankara, Turkey

4- Department of Biomedical Engineering, Rutgers University, 599 Taylor Rd., Piscataway, NJ 08854

*corresponding authors: (berkusta@gmail.com, ayse.asatekin@tufts.edu)

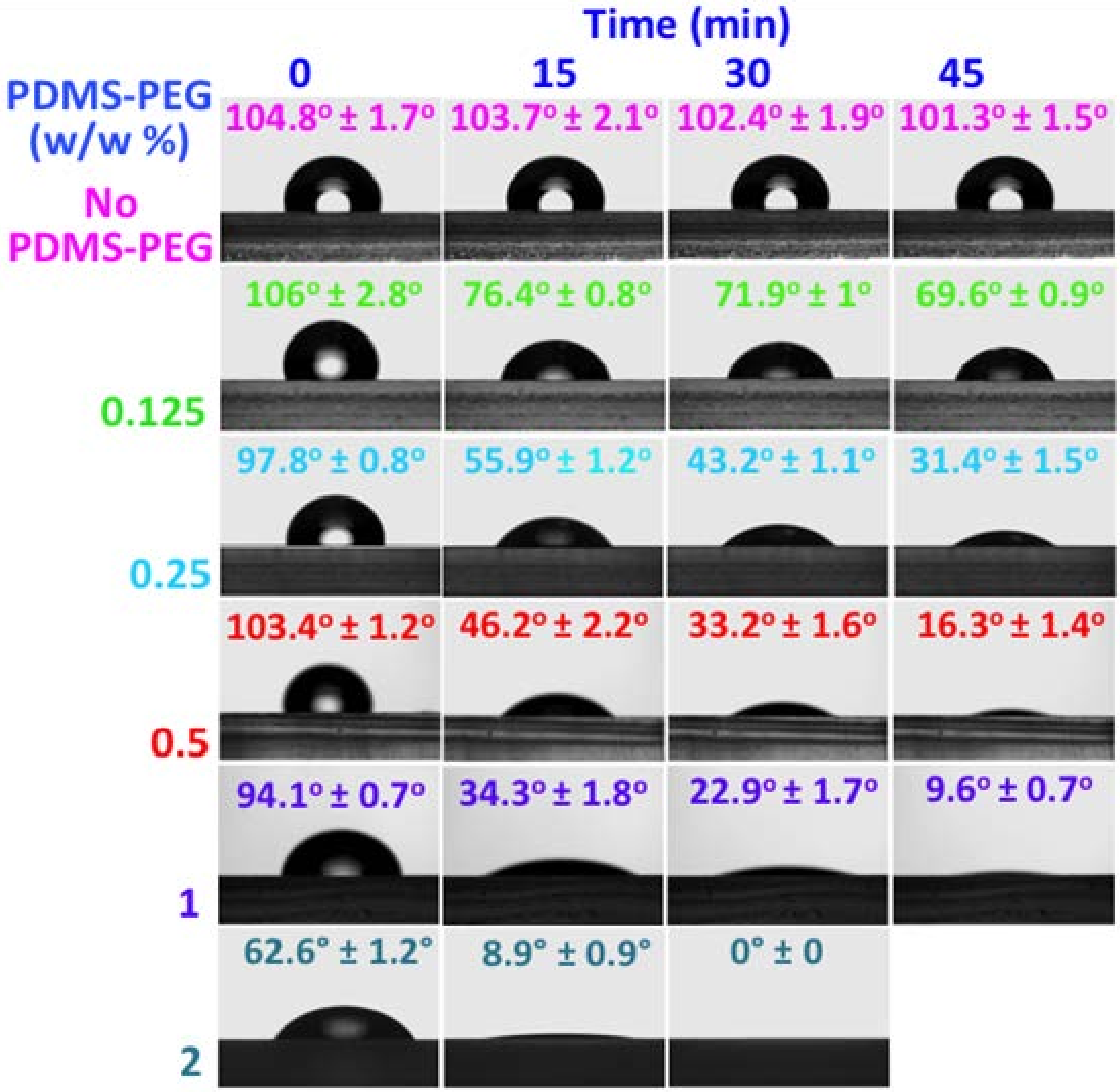

Fig. S1. PDMS with PDMS-PEG BCP additives dramatically reduces hydrophobicity. Variation of WCA of PDMS with no PDMS-PEG additives and with 0.125-2 (w/w %) PDMS-PEG additives in time. The data are shown as the mean ± SD (n=3).

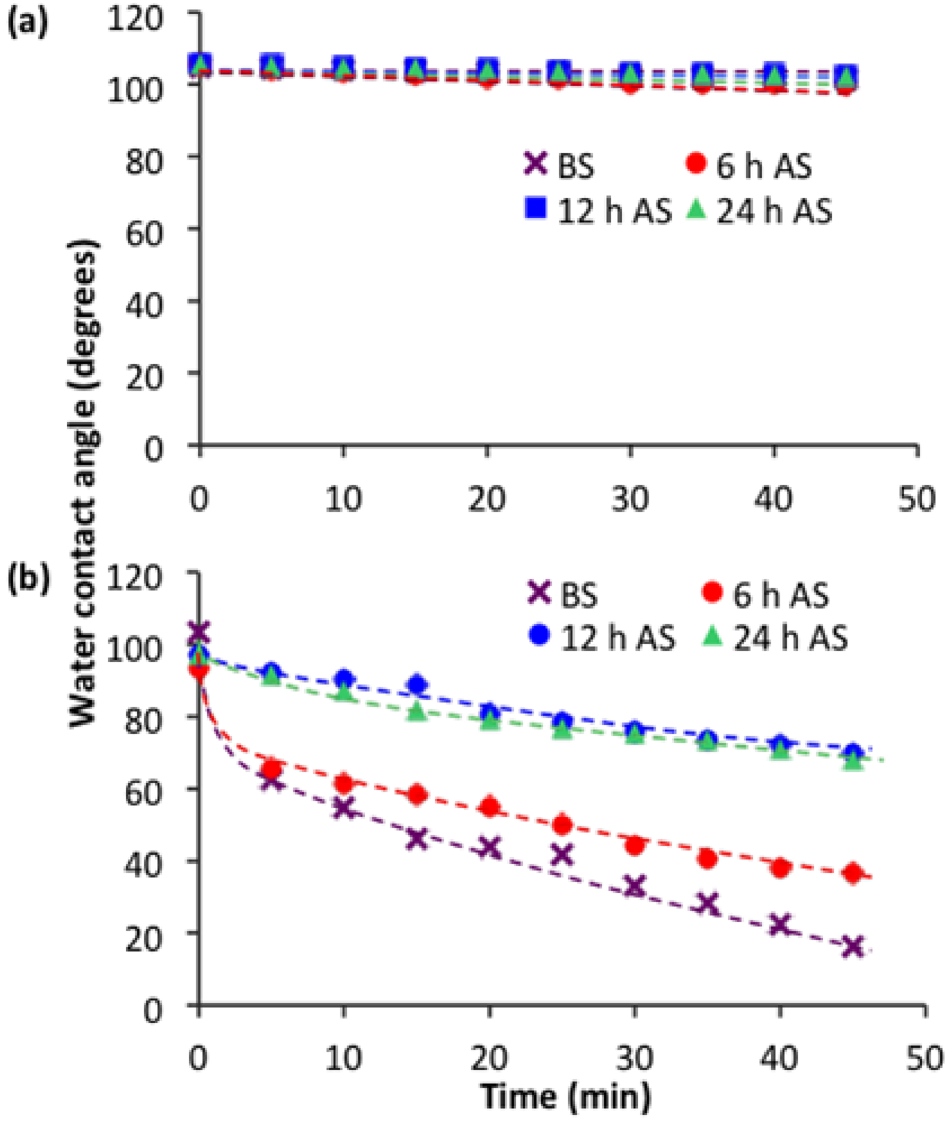

Fig. S2. The hydrophilicity of samples after 6, 12 and 24 h IPA soaking. WCA of (a) PDMS with no PDMS-PEG and (b) PDMS with 0.5% PDMS-PEG BCP additive after soaking the sample in IPA for 6, 12 and 24 hours. BS: Before IPA soaking, AS: After IPA soaking. The data are shown as the mean ± SD (n=3).

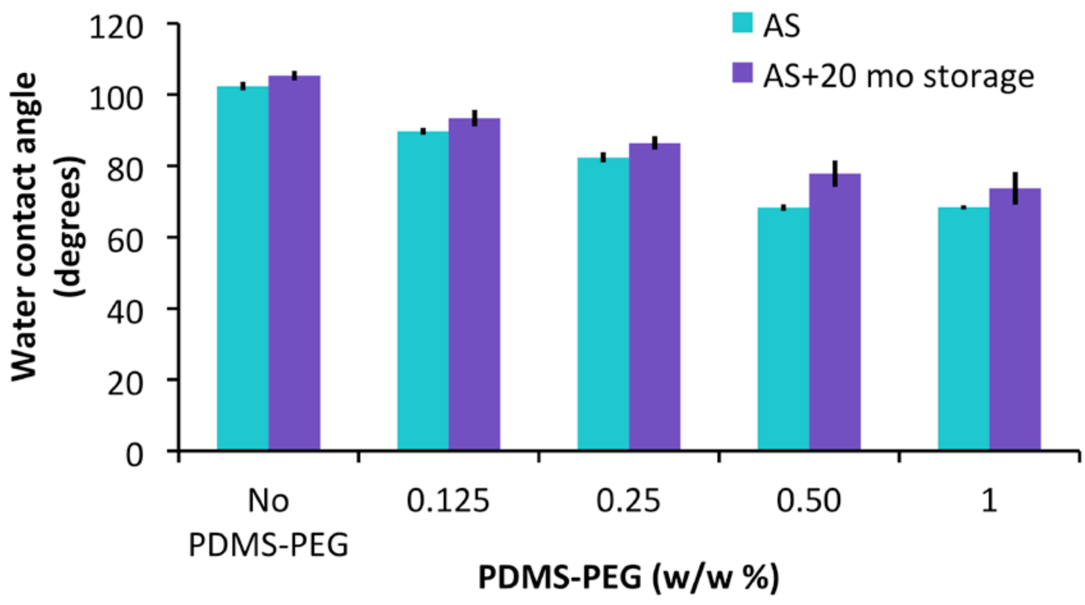

Fig. S3. Final (t=45 min) WCAs for different PDMS-PEG BCP additive ratios after IPA soaking, before and after 20 months of storage, show the stability of the modified materials. AS: A day after IPA soaking, AS+20 mo storage: After IPA soaking and 20 mo storage. The data are shown as the mean ± SD (n=3).

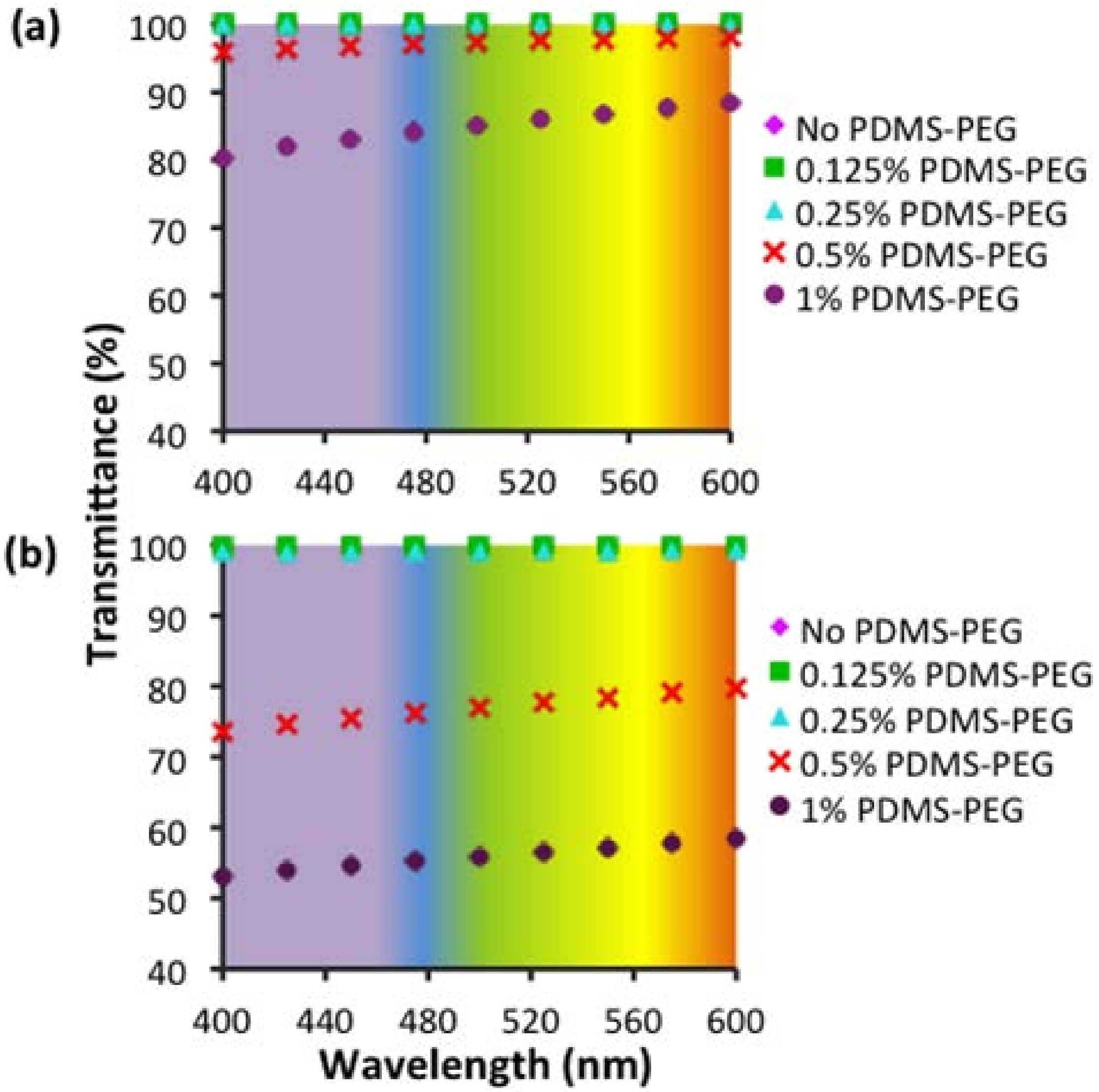

Fig. S4. The optical transmittence of PDMS with PDMS-PEG BCP additives between 400-600 nm. (A) before IPA soaking and (B) after IPA soaking were evaluated. Transparency of PDMS with 0.5% and 1% PDMS-PEG decreased after IPA soaking.

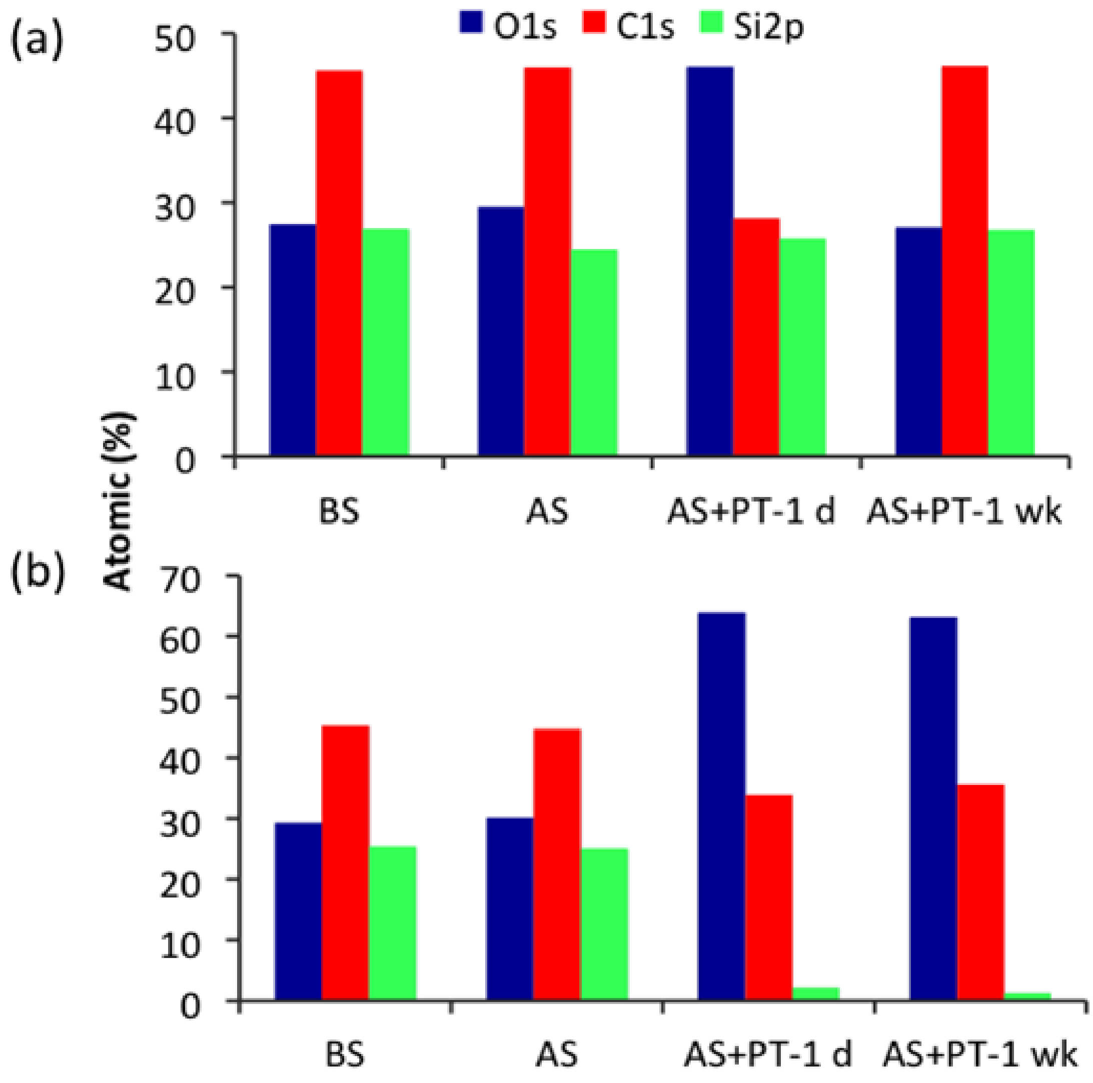

Fig. S5. Elemental surface compositions 0.25% PDMS-PEG confirm the PEG molecules on the surface. Wide scan XPS spectrum of (a) PDMS with no PDMS-PEG BCP (b) PDMS with 0.25% PDMS-PEG BCP. The survey scan was performed before IPA soaking (BS), after IPA soaking (AS), after IPA soaking and 1 day after plasma treatment (AS+PT-1 d), after IPA soaking and 1 week after plasma treatment (AS+PT-1 wk).

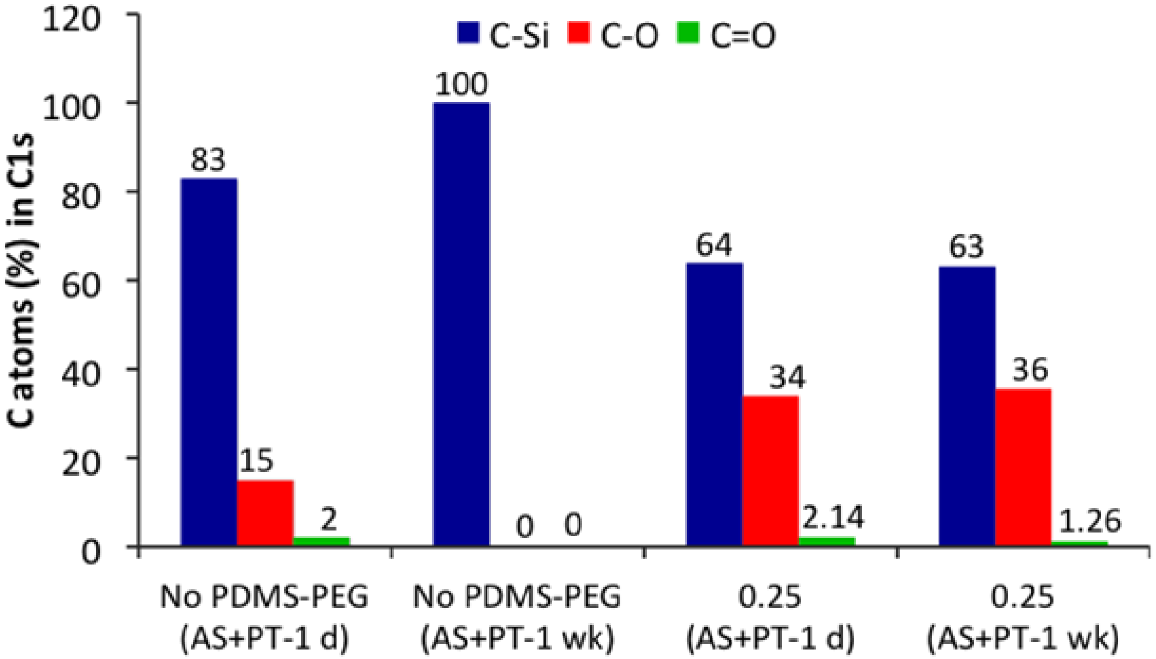

Fig. S6. Percentage of carbon atoms as C–Si, C–O and C=O, calculated by the deconvolution of peaks in the high resolution C1s XPS spectra of PDMS with no PDMS-PEG and PDMS with 0.25% PDMS-PEG BCP. The high resolution C1s spectra were measured after IPA soaking and 1 day after plasma treatment (AS+PT-1 d) and after IPA soaking and 1 week after plasma treatment (AS+PT-1 wk).

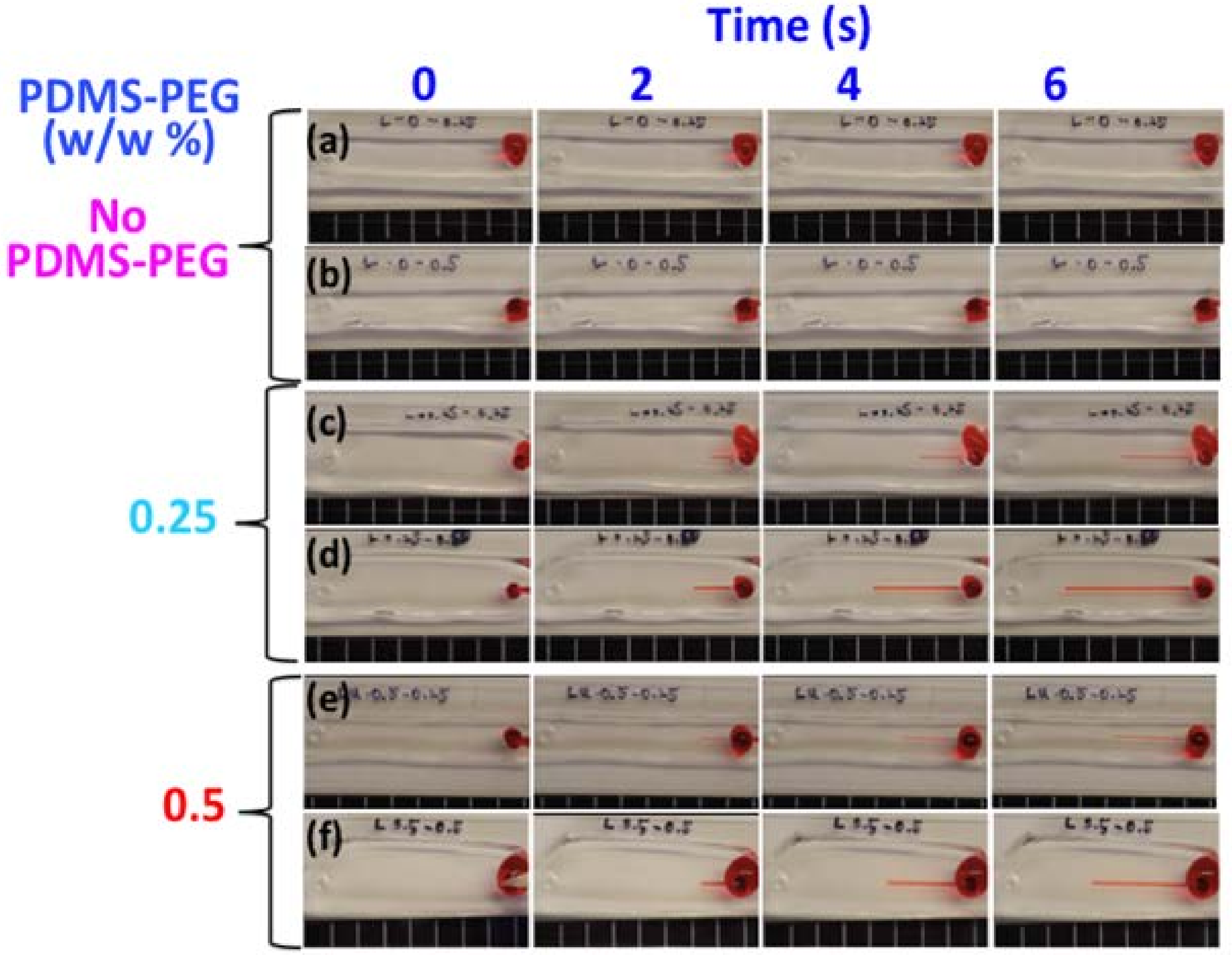

Fig. S7. Capillary-driven flow of hydrophilic PDMS channels with aqueous solutions. For this purpose PDMS with no PDMS-PEG and PDMS with 0.25% and 0.5% PDMS-PEG BCP were utilized. 0.25 mm ((a), (c), (e)) and 0.5 mm ((b), (d), (f)) channel widths were investigated.
